# Competition demands of elite wheelchair basketball athletes assessed with local positioning system and inertial measurement units

**DOI:** 10.3389/fspor.2026.1873335

**Published:** 2026-07-13

**Authors:** Leanne Snyder, Paul S. R. Goods, Brook Galna, Jeremiah J. Peiffer, Aaron Balloch, Peter Peeling, Martyn J. Binnie, Brendan R. Scott

**Affiliations:** 1 Physical Activity, Sport, and Exercise (PHASE) Research Group, School of Allied Health (Exercise Science), Murdoch University, Perth, WA, Australia; 2 Western Australian Institute of Sport, Perth, WA, Australia; 3 Parkinson School of Health Sciences and Public Health, Loyola University Chicago, Chicago, IL, United States; 4 Centre for Healthy Aging, Health Futures Institute, Murdoch University, Perth, WA, Australia; 5 School of Human Sciences, University of Western Australia, Perth, WA, Australia

**Keywords:** disability sport, movement demands, paralympic sport, physical demands, time-motion analysis

## Abstract

**Introduction:**

Understanding competition demands in elite wheelchair basketball (WCB) is essential for designing effective training programs given the range of impairments represented within teams. The aim was to examine movement demands in elite WCB by sex and Para-sport classification.

**Methods:**

Inertial measurement units (IMU) and local positioning system (LPS) data were collected during international series involving the Australian national WCB teams. The dataset included observations from 12 female athletes across five games and 14 male athletes across three games. Athletes were classified as either high-point (≥3.0; *n* = 5 females and *n* = 10 males) or low-point (≤2.5; *n* = 7 females and *n* = 4 males).

**Results:**

Relative competition demands (accelerations ≥1.0 m·s⁻^2^; high-intensity ≥2.0 m·s⁻^2^) were broadly similar across movement variables between female (81.4 m·min⁻¹, 9.3 accelerations·min⁻¹, 8.7 decelerations·min⁻¹, and 27.0 rotations·min⁻¹) and male athletes (83.9 m·min⁻¹, 9.5 accelerations·min⁻¹, 8.9 decelerations·min⁻¹, and 25.4 rotations·min⁻¹); however, male athletes performed more high-intensity accelerations and decelerations than females. Classification effects included: high-point female athletes recorded 8.6% higher peak speed (*p* < 0.001), 12.4% more accelerations (*p* = 0.014), and 12.0% more decelerations (*p* = 0.017) than low-point females, while low-point females demonstrated greater high-intensity acceleration rates (*p* = 0.002). In males, high-point athletes demonstrated higher mean (5.3%, *p* = 0.038) and peak speed (5.1%, *p* = 0.024) than low-point athletes.

**Discussion:**

These findings highlight similar movement demands between sexes, with differences emerging in the most demanding efforts and across classification groups. Training approaches may benefit from consideration of classification-relevant physical qualities, particularly high-intensity propulsion and braking capacities.

## 1 Introduction

Knowledge of competition demands is fundamental for designing sport-specific training programs, minimizing injury risk, and assisting with talent identification (1). Determining the movement demands of training and competition has been widely facilitated by global positioning system (GPS) technology; however, its inability to function indoors presents challenges for sports such as wheelchair basketball (WCB). Consequently, alternative solutions have been employed in WCB, including video analysis (1), magnetic reed-switch devices (2), miniaturized data loggers (3), inertial measurement units (IMUs) (108), and local positioning systems (LPSs) (4). Of these, only IMUs and LPSs have demonstrated promising validity for capturing multidirectional, high-speed movements in wheelchair court sports (LPS CV = 0.2%–1.6%, IMU ICC = 0.86–0.94) (4, 5). Though capable of capturing displacement and speed indoors, LPSs are less commonly used due to their high costs, extensive set-up, and lack of portability; therefore, displacement and speed data are limited in WCB. While IMUs are both practical and accessible, integrating LPSs (where feasible) may offer a more comprehensive overview of competition demands in WCB.

Few studies have quantified the movement demands of WCB competition, and many early investigations relied on technologies with important methodological limitations. Initial studies using miniaturized data loggers and magnetic reed-switch devices estimated that athletes covered ∼2.7–4.8 km per game at average speeds of 1.5–2.0 m·s⁻¹ (2, 3, 6); however, these approaches lacked validation, were inaccurate at higher speeds, or extrapolated data from limited playing time. More recent work using IMUs demonstrated that international-level athletes rotate faster than national-level athletes during competition, suggesting rotational speed may be an important indicator of WCB physical performance (7). Video-based analyses have further shown that elite WCB competition involves frequent gliding, rotational movements, and short-duration activity transition (1, 8). Together, these findings highlight the complex multidirectional demands of WCB competition, while also emphasizing the need for more comprehensive time-motion analyses using validated technologies such as IMUs and LPSs.

Further to competition level, movement demands specific to Para-sport classification remain poorly understood. To compete at the international level, athletes must meet specific minimum impairment criteria established by the international wheelchair basketball federation (IWBF) (9, 10), which classifies athletes on a scale from 1.0 (greater impairment impact) to 4.5 (lesser impairment impact). Existing evidence suggests that, compared with low-point athletes, high-point athletes tend to score more points, perform more rebounds and assists, demonstrate superior speed and change-of-direction performance in field testing (11, 12), and perform faster linear and rotational speeds in competition (7, 13). However, objective in-competition movement demand profiles quantified using sensor-based technologies remain limited across Para-sport classification groups, restricting the development of classification-specific training and performance strategies.

In non-disabled team sports, movement demands such as distance, speed, and change of direction typically decline across quarters, indicating progressive fatigue as matches unfold (14). Similar temporal trends have been reported in wheelchair rugby, where low-point athletes show more pronounced fatigue effects during games (15, 16). For instance, average speed (−19.1% in low-point and −10.1% in high-point athletes; *p* < 0.001) and total distance (−9.9% vs. −4.2%; *p* < 0.05) declined from the first to the second half, with larger reductions observed in low-point athletes (15, 16). These findings suggest classification-dependent performance reduction patterns may also exist in WCB, yet the influence of game quarter on WCB competition demands remains underexplored.

Finally, the existing WCB studies report male or combined male and female (2, 3, 6) movement demands, leaving a gap in our understanding of the competition demands for elite female WCB athletes. Although female and male athletes often train together in WCB for practical purposes, they do not compete together internationally. Therefore, our current knowledge lacks nuanced insights into how the demands of competition may differ between sexes, which could be critical for developing tailored training and performance strategies.

Current literature is limited in explaining the demands of WCB competition, with existing knowledge gained from some of the published work using unreliable devices (2, 3), combined female and male data (2, 6), or no consideration of athlete classification (1). Therefore, the competition demands of elite WCB are not yet fully understood. Accordingly, the aims of this study were to describe and compare how movement demands during elite WCB competition differed by athlete classification (low- and high-point athletes), and sex (females and males) using data derived from athlete-secured IMUs and an LPS. We hypothesized that males would perform more movements at a higher intensity compared to female athletes. Additionally, we expected high-point athletes to exhibit greater movement intensity than low-point athletes due to their less severe physical impairments.

## 2 Materials and methods

### 2.1 Experimental approach to the problem

Data were collected from the female and male Australian national WCB teams. Speed, distance, and acceleration data were collected from the LPS, and rotational movement demands were collected from IMUs. A total of three male games were included in the analysis from an international series in July 2022, and a total of five female games were included from an international series in September 2022. Both international series were performed in preparation for the March 2023 Asia-Oceania Zone Qualifier tournament. Athletes' movement data were included in the analysis if they recorded more than 10 min of playing time for the game. As a result, a total of 54 athlete-game observations were recorded from 26 unique athletes across the eight games.

### 2.2 Participants

Twenty-six national team athletes participated in this study (a breakdown of athlete categorization and sex is provided in Table 1). Athletes were recruited from the Australian national female and male wheelchair basketball teams competing in the international series. Athlete classifications ranged from 1.0 to 4.5, including half-point classifications. Female athletes were aged 25.2 ± 2.9 and male athletes 31.0 ± 4.2 y. All athletes were informed about the research protocol, requirements, benefits, and risks, and their written consent was obtained before study commencement. Ethical approval was granted by the Institutional Human Research Ethics Committee (2022/055).

**Table 1:** Overview of the Australian national wheelchair basketball squad participants grouped by athlete classification group and sex. Athletes were grouped according to the International Wheelchair Basketball Federation disability classification system (scale: 1.0–4.5) as low- (≤2.5, greater impairment impact) and high- (≥ 3.0, lesser impairment impact) point athletes.

**Classification Group**	**Females (*n*)**	**Males *(n*)**	**Total athletes (*n*)**
High	5	10	15
Low	7	4	11
Total	12	14	26

### 2.3 Procedures

Athletes were fitted with a wearable tracking sensor (Vector T7, Catapult Sports, Melbourne, Australia) containing an LPS sensor (10 Hz sampling frequency) and IMU (tri-axial accelerometer, gyroscope, and magnetometer at 100 Hz sampling frequency) connected wirelessly to the LPS (Clearsky, Catapult Sports, Melbourne, Australia). Sensors were secured to the athletes in a fitted pouch built into a sport vest (upper back between the scapulae) to ensure a close fit to the body without restricting movement, as athlete-secured sensors capture trunk-related movements that may not be detected by wheelchair-secured approaches, which may be particularly relevant for distinguishing classification-related movement patterns (17). The LPS consisted of a pre-installed network of 20 anchors within the stadium positioned above the court which tracked the sensors' positional movement data using ultra-wideband technology. This specific LPS has demonstrated validity for movement analysis with indoor team sport applications for linear and multidirectional tasks (mean positional difference = 0.21 ± 0.13 m, mean average speed difference <0.07 ± 0.17 m ·s^−1^, and mean instantaneous speed >3 m·s^−1^ difference at 0.68 ± 0.58 m·s^−1^) against a gold standard reference infrared light-based camera system (18). The anchor setup used to collect this data closely reflects the optimal indoor ClearSky configuration (18). Accompanying proprietary software (Openfield, Catapult Sports, Melbourne, Australia v1.22.0) was used to trim the game data to include only active playing time (i.e., with bench time and breaks between quarters excluded).

From each game observation, spatiotemporal and accelerometer-derived variables were analyzed and extracted using Matlab (Mathworks, v2024a). Speed and distance data [total distance, mean speed (total distance covered ÷ total active game time), peak speed], acceleration data (accelerations count, decelerations count, and acceleration RMS [root mean square (magnitude of all acceleration data)] were extracted from the LPS sensor. Acceleration RMS was calculated to provide a continuous measure of movement intensity, reflecting both the magnitude and frequency of accelerations. RMS has been widely used in biomechanics as a robust measure of signal power over time (19).

Acceleration events were identified for all acceleration peaks ≥1 m·s⁻^2^ and deceleration events were calculated similarly using a threshold of nadirs ≤ −1 m·s⁻^2^. In addition, high-intensity acceleration and deceleration variables were derived from in-game performance data for all acceleration peaks ≥2 m·s⁻^2^ and deceleration events were calculated similarly using a threshold of nadirs ≤ −2 m·s⁻^2^. To be recorded as an event, the effort had to be sustained above threshold for at least 100 ms (one 10 Hz frame), and a minimum 500 ms inter-event interval was used to avoid double-counting. This approach is consistent with methodological recommendations for minimizing artefactual event detection in team sport tracking (20).

Acceleration and deceleration thresholds were selected using a data-driven sensitivity analysis, where acceleration and deceleration counts were examined across a range of thresholds (0.1–3.0 m·s⁻^2^). The results of the sensitivity analysis are provided in the Supplementary Materials (Supplementary Figure S1 and Supplementary Table S1). Thresholds above 2.0 m·s⁻^2^ substantially underestimated event counts, while thresholds below 1.0 m·s⁻^2^ resulted in large counts likely reflecting non-meaningful movement noise. A threshold of |1.0 m·s⁻^2^| was therefore selected as the minimum meaningful threshold. To capture higher-intensity efforts, a secondary threshold of |2.0 m·s⁻^2^| was defined based on preliminary analyses anchored to the distribution of peak accelerations observed during competition; detailed methodology is provided elsewhere (17).

Rotational event game data (rotation count, mean and peak rotational velocity) were extracted from the IMU sensor using gyroscope data about the longitudinal axis of the sensor (yaw). A zero-lag second-order low-pass Butterworth filter with a 20 Hz cut-off was applied (7). Rotational events were calculated for all events ≥10°·s⁻¹ based on previous WCB research (7), and a rotational displacement of ≥30°, adapted from existing change-of-direction thresholds used in court sports (21). A minimum event duration of ≥0.2 s was selected based on a sensitivity analysis demonstrating that longer duration thresholds substantially underestimated rotation counts, particularly for athlete-secured sensors (Supplementary Figure S2) (17).

### 2.4 Statistical analyses

Data normality was assessed using the Shapiro–Wilk test. Relative movement demands were calculated by dividing the total absolute counts by the athlete's minutes played, resulting in the number of events per minute. A linear mixed model was used to compare absolute game data between female and male athlete games. Sex was set as the fixed factor, and athletes were included as random intercepts. Separate models were then run for females and males to evaluate the effects of classification group and game quarter on movement demands within each sex, with classification group and quarter of game set as fixed factors and athletes as random intercepts. Two-way interactions between the fixed factors (classification x quarter) were included. Bonferroni *post-hoc* tests were applied for pairwise comparisons where significant main effects and interactions were observed to control for multiple comparisons within each family of tests. Standardized effect sizes (Hedges' g) were calculated for significant sex and classification comparisons.

**Figure 1:**
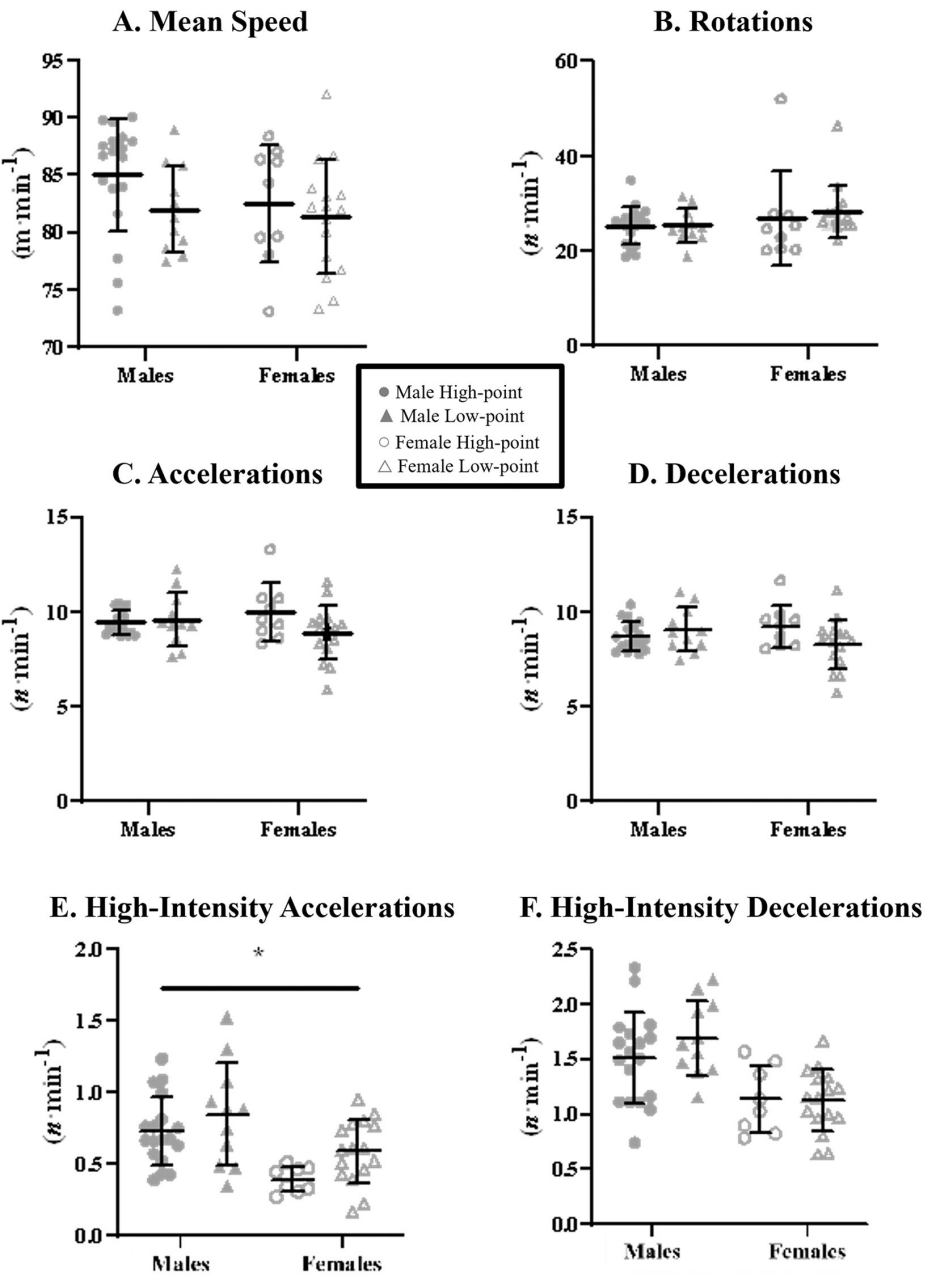
Relative movement demands by sex and classification. Mean speed **(A)**, accelerations **(C)**, decelerations **(D)**, and rotations **(B)** per minute showed no significant main effects of sex or classification and no interaction. High-intensity accelerations per minute were higher in males than females (*p* = 0.004) **(E)**. The overall classification effect for high-intensity accelerations was not significant (*p* = 0.058); within females, low-point >high-point (*p* = 0.002), with no classification effect in males. High-intensity decelerations per minute were higher in males than females (*p* < 0.001) **(F)**. No sex ×  classification interactions were observed. Significant effects are indicated with *.

Relative game demands were analysed with sex and classification included as fixed factors and athletes as a random intercept (Figure 1). Due to a violation of normality in the high classification group for athlete rotations per minute (Figure 1), a generalised estimating equations model was performed. A Gamma distribution with a log link function was used to account for the skewed nature of the data. The fixed effects included sex, classification group, and their interaction; the athlete was set as the subject factor for repeated measures. Due to a violation of normality in the female high-point group for accelerations count, decelerations count, and rotations count (Figure 1), a generalised estimating equations model was also employed for this output. A negative binomial with a log link function was used to account for the over dispersed nature of the data. The fixed effects included classification group, and the athlete was set as the subject factor for repeated measures.

All statistical analyses were performed using SPSS (v24; IBM, Chicago, Illinois), with significance set at *p* ≤ 0.05. Unless otherwise specified, data are presented as mea*n* ± standard deviation.

## 3 Results

Descriptive statistics for absolute and relative movement demands for female and male athletes are presented in Table 2. Absolute outputs were higher for female athletes, reflecting greater average playing time. When scaled relative to playing time, total distance, accelerations, decelerations, and rotations were similar between sexes. However, male athletes recorded higher rates of high-intensity accelerations and decelerations.

**Table 2:** Descriptive statistics (mean ± standard deviation) of absolute and relative game outputs for five female and three male elite wheelchair basketball games.

	**Females**		**Males**	
Output	Absolute values	Relative to playing time (units·min^−1^)	Absolute values	Relative to playing time (units·min^−1^)
Playing time (min)	35.2 ± 15.5		26.5 ± 10.1	
Total distance (m)	2,899 ± 1,349	81.4 ± 4.6	2,228 ± 837	83.9 ± 4.7
Acceleration count (*n*)	324 ± 153	9.3 ± 1.4	253 ± 101	9.5 ± 1.0
Deceleration count (*n*)	302 ± 141	8.7 ± 1.2	236 ± 95	8.9 ± 0.9
High-intensity accelerations (*n*)	18.6 ± 11.7	0.5 ± 0.2	19.9 ± 9.7	0.8 ± 0.3
High-intensity decelerations (*n*)	41.0 ± 23.0	1.1 ± 0.3	41.3 ± 17.9	1.6 ± 0.4
Rotations (*n*)	914 ± 351	27.0 ± 6.9	651 ± 203	25.4 ± 3.8

Descriptive statistics for female relative movement demands are presented in Table 3. High-point female athletes demonstrated greater peak speed (*g* = 0.58), accelerations per minute (*g* = 0.56), and decelerations per minute (*g* = 0.53) compared to low-point female athletes. Mean speed was significantly higher in Quarter 2 when compared to Quarter 1, regardless of classification. Low-point female athletes demonstrated a greater count of high-intensity accelerations (*g* = 0.57), regardless of quarter. For high-intensity decelerations, counts in Quarter 3 were significantly lower compared to Quarter 1 and Quarter 2.

**Table 3:** Mean ± SD of movement demands for speed and distance, acceleration, and rotational movement demands in quarters 1–4 (Q1–4) for 12 high- and low-point female athletes over five games. Demands are reported relative to playing time within each quarter.

**Output**	**Q1**	**Q2**	**Q3**	**Q4**	**Total Game**	**Classification Group (*p*)**	**Quarter (*p*)**	**Interaction (*p*)**
Playing time (min)								
High-point	12.6 ± 3.6	10.8 ± 3.6	11.7 ± 3.2	10.0 ± 4.8	40.0 ± 16.1	0.317	0.475	0.472
Low-point	8.9 ± 4.0	7.4 ± 3.7	8.9 ± 4.5	9.4 ± 4.8	32.5 ± 15.5			
Mean speed (m·min^−1^)^b^								
High-point	78.9 ± 6.7	85.7 ± 6.6	85.3 ± 7.3	80.1 ± 6.6	82.5 ± 5.1	0.909	**0**.**033**	0.651
Low-point	79.5 ± 5.8	84.8 ± 6.4	82.5 ± 10.5	82.6 ± 5.1	81.3 ± 4.9			
Peak speed (m·s^−1^)								
High-point^a^	3.8 ± 0.3	3.8 ± 0.2	3.7 ± 0.2	3.7 ± 0.2	3.8 ± 0.3	**<0** **.** **001**	0.998	0.831
Low-point	3.5 ± 0.3	3.5 ± 0.3	3.5 ± 0.3	3.5 ± 0.3	3.5 ± 0.3			
Accelerations (*n·*min^−1^)								
High-point^a^	9.5 ± 1.0	10.3 ± 1.6	9.9 ± 1.3	9.5 ± 1.8	10.0 ± 1.5	**0**.**014**	0.444	0.424
Low-point	9.4 ± 1.6	9.3 ± 1.6	8.2 ± 1.7	8.9 ± 1.8	8.9 ± 1.4			
Decelerations (*n·*min^−1^)								
High-point^a^	9.2 ± 0.9	9.3 ± 1.4	9.4 ± 0.9	8.5 ± 0.8	9.3 ± 1.1	**0**.**017**	0.674	0.484
Low-point	8.7 ± 1.9	8.5 ± 1.5	7.8 ± 1.4	8.4 ± 1.6	8.3 ± 1.3			
High-intensity accelerations (*n·*min^−1^)								
High-point^a^	0.4 ± 0.1	0.4 ± 0.2	0.5 ± 0.2	0.4 ± 0.3	0.4 ± 0.1	**0**.**002**	0.598	0.185
Low-point	0.6 ± 0.2	0.6 ± 0.4	0.5 ± 0.3	0.6 ± 0.2	0.6 ± 0.2			
High-intensity decelerations (*n·*min^−1^)^c^^,^^d^								
High-point	1.2 ± 0.3	1.4 ± 0.5	1.0 ± 0.3	1.1 ± 0.5	1.2 ± 0.3	0.993	**<0**.**001**	0.480
Low-point	1.2±0.5	1.4 ± 0.5	0.5 ± 0.3	0.6 ± 0.2	0.6 ± 0.2			
Mean angular velocity (*°·*s^−1^)								
High-point	95.2 ± 5.6	97.7 ± 5.9	94.0 ± 7.0	95.2 ± 7.4	95.3 ± 6.4	0.223	0.708	0.943
Low-point	110.0 ± 27.1	111.3 ± 22.1	102.1 ± 10.5	107.8 ± 22.0	107.9 ± 21.3			
Peak angular velocity (*°·*s^−1^)								
High-point	462.9 ± 50.2	489.9 ± 108.9	477.7 ± 40.8	469.4 ± 116.4	474.3 ± 82.7	0.144	0.977	0.860
Low-point	544.3 ± 130.6	530.7 ± 130.1	516.7 ± 111.0	558.0 ± 193.0	537.9 ± 142.0			
Mean angular displacement (*°*)								
High-point	69.8 ± 3.1	68.6 ± 4.1	68.9 ± 4.2	65.7 ± 4.2	67.9 ± 3.5	0.126	0.247	0.750
Low-point	77.4 ± 10.8	75.8 ± 9.6	78.6 ± 8.9	76.6 ± 9.1	76.9 ± 9.4			

Significant main effects are indicated in bold text.

a Significantly different to low-point athletes.

b Quarter 1 is significantly different to Quarter 2.

c Quarter 3 is significantly different to Quarter 1.

d Quarter 3 is significantly different to Quarter 2.

Descriptive statistics for the relative movement demands of male athletes are presented in Table 4. High-point male athletes had significantly higher mean speed (g = 0.59) and peak speed (g = 0.54) than low-point athletes. Peak speeds were higher in Quarter 1 when compared to Quarter 2 in male athletes, regardless of classification. For high-intensity decelerations, a significant main effect of quarter was observed, although *post-hoc* comparisons did not reveal differences between individual quarters.

**Table 4:** Mean ± SD of movement demands for speed and distance, acceleration, and rotational movement demands in quarters 1–4 (Q1–4) for 12 high- and low-point male athletes over three games. Demands are reported relative to playing time within each quarter.

**Effect**	**Q1**	**Q2**	**Q3**	**Q4**	**Total Game**	**Classification Group (*p*)**	**Quarter (*p*)**	**Interaction (*p*)**
Playing time (min)^b^^,^^c^								
High-point^a^	8.0 ± 2.1	8.0 ± 1.9	7.5 ± 2.2	8.5 ± 2.8	23.8 ± 7.9	**0**.**006**	**0**.**019**	**0**.**003**
Low-point	11.8 ± 1.6	7.9 ± 2.8	12.1 ± 3.3	9.2 ± 3.1	31.0 ± 12.0			
Mean speed (m·min^−1^)								
High-point^a^	87.2 ± 8.5	85.3 ± 8.4	85.5 ± 4.7	86.3 ± 8.9	86.1 ± 7.6	**0**.**038**	0.362	0.802
Low-point	85.2 ± 5.4	79.1 ± 5.8	82.3 ± 3.2	81.6 ± 7.0	81.8 ± 6.1			
Peak speed (m·s^−1^)^b^								
High-point^a^	4.1 ± 0.2	4.1 ± 0.3	4.1 ± 0.2	4.2 ± 0.2	4.1 ± 0.2	**0**.**024**	**0**.**015**	0.281
Low-point	4.0 ± 0.1	3.7 ± 0.3	3.8 ± 0.1	3.9 ± 0.2	3.9 ± 0.2			
Accelerations (*n·*min^−1^)								
High-point	9.3 ± 1.4	9.3 ± 1.1	9.5 ± 0.9	9.7 ± 0.8	9.4 ± 0.6	0.530	0.353	0.198
Low-point	10.5 ± 1.2	9.2 ± 1.4	10.0 ± 1.3	9.2 ± 1.4	9.6 ± 1.4			
Decelerations (*n·*min^−1^)								
High-point	8.4 ± 1.0	8.5 ± 1.3	8.8 ± 1.0	9.1 ± 1.0	8.7 ± 0.8	0.425	0.170	0.394
Low-point	9.3 ± 0.9	8.6 ± 1.0	9.7 ± 1.6	9.1 ± 1.3	9.1 ± 1.2			
High-intensity accelerations (*n·*min^−1^)								
High-point	0.4 ± 0.2	0.4 ± 0.1	0.4 ± 0.1	0.4 ± 0.2	0.4 ± 0.1	0.297	0.815	0.640
Low-point	0.4 ± 0.2	0.3 ± 0.1	0.3 ± 0.1	0.3 ± 0.2	0.3 ± 0.1			
High-intensity decelerations (*n·*min^−1^)								
High-point	1.3 ± 0.4	1.2 ± 0.4	1.1 ± 0.3	1.3 ± 0.4	1.2 ± 0.3	0.135	**0**.**044**	0.170
Low-point	1.1 ± 0.5	1.1 ± 0.4	1.0 ± 0.3	1.0 ± 0.3	1.0 ± 0.3			
Accelerations root mean square (*m·*s^−2^)								
High-point	0.70 ± 0.05	0.69 ± 0.04	0.70 ± 0.03	0.73 ± 0.02	0.71 ± 0.03	0.429	0.216	0.151
Low-point	0.73 ± 0.03	0.70 ± 0.03	0.73 ± 0.05	0.70 ± 0.05	0.71 ± 0.03			
Rotations (*n·*min^−1^)								
High-point	24.3 ± 4.5	24.0 ± 3.7	24.3 ± 3.6	25.2 ± 4.2	24.4 ± 3.9	0.739	0.822	0.105
Low-point	24.1 ± 2.9	25.9 ± 3.8	24.3 ± 3.3	24.8 ± 4.2	24.9 ± 3.8			
Mean angular velocity (°·s^−1^)								
High-point	98.3 ± 12.5	100.6 ± 10.2	99.4 ± 10.6	101.8 ± 14.0	100.0 ± 11.6	0.317	0.894	0.922
Low-point	116.7 ± 8.6	113.7 ± 11.8	117.1 ± 9.6	111.9 ± 12.3	114.4 ± 10.7			
Peak angular velocity (°·s^−1^)								
High-point	486.5 ± 124.3	512.9 ± 93.9	489.5 ± 146.0	524.9 ± 171.0	502.7 ± 142.3	0.164	0.958	0.999
Low-point	669.7 ± 114.9	641.8 ± 239.5	669.1 ± 188.0	633.6 ± 189.0	650.5 ± 183.9			
Mean angular displacement (°)								
High-point	72.4 ± 10.1	74.4 ± 9.3	75.1 ± 9.7	72.9 ± 7.6	73.4 ± 8.5	0.389	0.199	0.346
Low-point	76.3 ± 3.2	74.0 ± 6.3	75.6 ± 3.5	77.2 ± 5.7	76.4 ± 4.7			

Significant main effects are indicated in bold text.

a Significantly different to low-point athletes.

b Quarter 1 is significantly different to Quarter 2.

c Quarter 2 is significantly different to Quarter 3.

Across the whole-game analyses relative to playing time (Figure 1), male athletes recorded higher high-intensity accelerations (0.79 ± 0.25 vs. 0.50 ± 0.16 events·min⁻¹; *p* = 0.004; ∼58% higher; g = 0.71) and high-intensity decelerations (1.61 ± 0.44 vs. 1.14 ± 0.26 events·min⁻¹; *p* < 0.001; ∼41% higher; g = 0.78) than females. The overall main effect of classification on high-intensity accelerations was not significant (*p* = 0.058). In sex-specific models, however, low-point females performed more high-intensity accelerations than high-point females (*p* = 0.002; g = 0.57), whereas no classification effect was observed in males (*p* = 0.297). No sex ×  classification interactions were present (*p* = 0.163 to 0.227).

## 4 Discussion

The aim of this study was to describe the movement demands of elite WCB competition according to athlete classification and sex, and to compare differences in these demands between groups. Overall, movement demands were similar in volume across sexes when expressed relative to playing time; however, they differed in intensity, with male athletes completing more high-intensity accelerations than females. Among males, high-point athletes demonstrated higher mean and peak speeds than low-point athletes, while in females, low-point athletes performed more high-intensity accelerations, and high-pointers showed greater peak speeds and more frequent acceleration and deceleration efforts. These findings suggest that overall movement demands are comparable between sexes, although differences in high-intensity efforts may warrant consideration. Mixed training may still be appropriate, provided that drills and load management reflect the physical capacities and movement demands of each classification group.

When analyzed relative to playing time, movement demands were similar between sexes. Compared with an earlier video-based analysis of male and female WCB athletes across nine games (8) the present study recorded roughly twice as many rotations and substantially more decelerations per game. This discrepancy likely reflects the greater sensitivity of IMU and LPS technologies for detecting short-duration movements, whereas the earlier video-based study did not specify thresholds for defining these actions, limiting direct comparison. The overall similarity in relative movement demands between sexes aligns with previous research using three IMUs per athlete, which also reported comparable results across most variables, except for lower average rotational acceleration in females. Instead, functional capacity linked to classification, such as trunk control, seating position, and reach height, appears to play a more prominent role in shaping movement profiles. When high-intensity thresholds of |2.0 m·s⁻^2^| for accelerations and decelerations were applied, male athletes performed more accelerations and decelerations than females. These differences may reflect nuances in how load is accumulated during competition but could also be influenced by contextual factors such as playing experience, physical strength, or tactical role, rather than inherent sex-based differences alone (22–24). However, these findings should be interpreted cautiously given the relatively small subgroup sizes and exploratory nature of some comparisons.

Classification effects were small when scaled to playing time but became more apparent when examining high-intensity movements. High-point athletes demonstrated greater movement capacity, including higher peak speeds, which may reflect their greater trunk mobility and higher seating positions, which enhance their ability to generate propulsion and perform short accelerations from a stationary position (25). High-point athletes generally assume post and center roles, where their higher seating position enables them to rebound and shoot over opponents (26, 27). However, seating position and wheelchair setup characteristics were not directly measured or controlled in the present study and may differ substantially even within classification groups. In contrast, low-point athletes often adopt deeper-seated positions that prioritize stability due to reduced trunk control, which tends to make them suited for guard roles centered on ball-handling, defensive coverage, and pick setting around the perimeter (28). While this configuration improves stability, it may limit high-intensity movement capacity (25). Interestingly, low-point female athletes demonstrated greater high-intensity acceleration counts than high-point athletes, despite reduced trunk control typically being associated with lower movement capacity. One possible explanation is that athletes with limited active trunk control may experience larger reactive trunk movements during rapid changes in wheelchair velocity. Consequently, sudden propulsion or braking actions may produce greater trunk motion, potentially contributing to the detection of more high-intensity acceleration events. However, trunk kinematics were not directly measured in the present study, and this interpretation remains speculative. Accordingly, the finding should be interpreted cautiously given the small subgroup sizes. Although wheelchair setup constraints are often non-modifiable to an extent for low-point athletes who require greater stability, coaches can still enhance performance by targeting upper-body strength and power (24) and pushing technique (23, 29, 30), which are modifiable characteristics that have been shown to improve speed and acceleration in this group. Targeted development of these characteristics may help support acceleration and speed performance across classification groups. Classification-based differences may reflect interactions between physical capabilities and tactical responsibilities (11). However, athlete roles and tactical strategies were not directly measured in the present study, and these interpretations should therefore be considered speculative.

The quarter-based analysis provided novel insight into how movement demands fluctuate across match play in elite WCB. Peak and mean speed showed only minor, non-significant changes across quarters. However, when high-intensity thresholds were applied, some temporal patterns were observed, particularly in low-point athletes, who demonstrated significant reductions in the number of high-intensity accelerations and decelerations across quarters. High-point athletes, however, showed no significant quarter-to-quarter differences in any variable. While this may reflect differences in functional capacity between classification groups, other contributing factors such as pacing strategies, playing time distribution, tactical roles, and contextual game demands may also have influenced these findings. Trunk control and fatigue responses were not directly measured in the present study and therefore these interpretations remain speculative. These findings are partly consistent with research in wheelchair rugby, where more substantial performance declines over quarters have been reported in low-point athletes (15). However, the changes observed in WCB were comparatively modest, which may reflect differences in impairment profiles, classification requirements, or tactical demands between the two sports. As one of the first investigations to report quarter-based WCB data, these findings should be interpreted cautiously, and further research involving larger samples, contextual match variables, additional games and teams is needed to better understand temporal trends across match play.

Previous research has shown that high-point athletes accumulate more visible game statistics, whereas low-point athletes may contribute through less commonly reported actions such as setting picks, reflecting different on-court roles (31). Our findings align with previous research that observed significantly faster linear speed performance in high-point athletes compared to low-point athletes in competition (7), and this was further supported in our high-intensity analyses, where high-point athletes demonstrated a greater number of high-intensity acceleration efforts. In contrast, we did not observe classification-based differences in rotational movements, despite previous reports of faster rotational performance in field tests for high-point athletes (7). Similar discrepancies between field and competition measures have been noted in other studies (32). Significant differences were observed between low- and high-point athletes, but these differences were more pronounced in field tests (32), where differences between low- and high-classified athletes were more pronounced in testing than in match play. This suggests that observed movement demands are shaped by tactical context as well as physical capacity.

Overall, this research provides novel insight into the competition demands of elite female and male WCB athletes; however, several limitations should be acknowledged. First, the small sample size, owing to recruitment from a single nation's female and male teams, limits the certainty of our interpretations and the generalizability of findings to other contexts. Additionally, although mixed modelling approaches were used to account for repeated observations within athletes, the relatively small number of games and exploratory subgroup analyses increase the potential for both Type I and Type II error. Accordingly, findings relating to classification- and quarter-specific effects should be interpreted cautiously and considered preliminary until replicated in larger and more diverse cohorts. Nevertheless, given the scarcity of research in this area and the emerging nature of the sport at a domestic level, this study represents one of the most comprehensive datasets currently available and provides preliminary evidence for extending objective monitoring into sub-elite settings. Second, movement demands are also likely influenced by contextual factors such as coaching strategies, team composition, and game situations (e.g., playing style, score margin, or phase of play), which were not captured in the present study. While the present study quantified external movement demands, these outputs should not be interpreted as direct measures of physiological or perceptual load. The physiological cost of movement may differ substantially between athletes depending on impairment characteristics, functional capacity, and movement efficiency. Future research integrating internal load measures such as heart rate, session-RPE, and contextual match variables may provide a more comprehensive understanding of competition demands in elite WCB. Finally, while we reported data on accelerations, decelerations, and rotations, standardized wheelchair-sport specific thresholds for these metrics have not yet been established. To address this, thresholds were derived using the data-driven sensitivity analyses described elsewhere (17), providing greater transparency and justification. These cut-offs provide an evidence-based starting point for future monitoring practices, but continued refinement across larger and more diverse cohorts will help ensure their robustness and applicability.

This study highlights that, while elite male and female WCB athletes experience broadly similar overall movement demands, differences emerge at higher intensities and between classification groups. High-point athletes moved significantly faster and performed more demanding propulsion efforts, which may reflect greater trunk mobility, wheelchair set up characteristics such as higher seating positions, and their typical on-court roles. These findings emphasize the need for training approaches that balance shared and classification-specific demands. Training together across classifications and sexes can foster tactical cohesion and versatility, reflecting the mixed line-ups used in competition. At the same time, targeted emphasis on classification-specific qualities, such as trunk stability and pushing technique in low-pointers or speed and power development in high-pointers, ensures that individual physical needs are addressed. Combining these approaches may best prepare athletes to meet both the collective and individual demands of elite WCB. Future research across larger and more diverse samples is needed to confirm these patterns and further refine training recommendations.

## Data Availability

The raw data supporting the conclusions of this article are available by request.
