# Supplementary material for: Competition demands of elite wheelchair basketball athletes assessed with local positioning system and inertial measurement units
Source: Front Sports Act Living. 2026 Jul 13;8:1873335. doi: 10.3389/fspor.2026.1873335 (PMC13402498; doi:10.3389/fspor.2026.1873335)
Supplement: Supplementary file 1 [file Supplementaryfile1.docx]

**Supplementary Table S1**. Mean acceleration event counts across thresholds for athlete-secured sensors derived from 54 elite wheelchair basketball athlete-games.

| **Threshold (**m·s⁻²) | **Acceleration events**  **per game, mean (range)** |
| --- | --- |
| 0.5 | 687 (277, 1,417) |
| 1.0 | 288 (100, 618) |
| 1.5 | 90 (18, 186) |
| 2.0 | 20 (3, 54) |
| 2.5 | 4 (0, 16) |
| 3.0 | 1 (0, 8) |


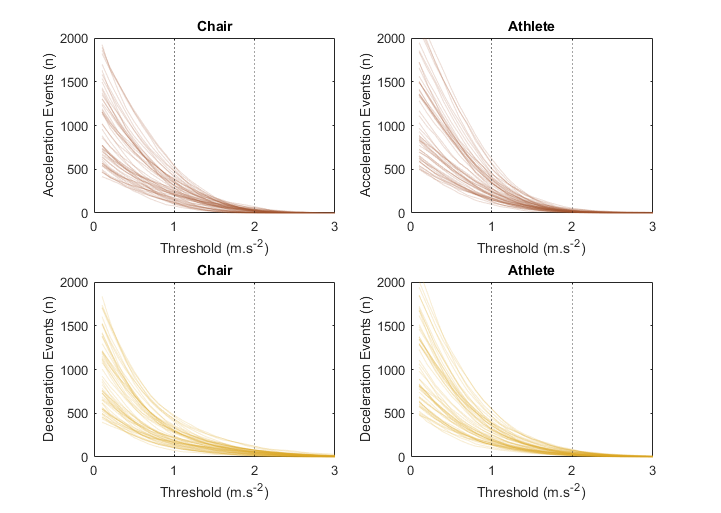


**Supplementary Figure S1.** Sensitivity curves showing the decline in acceleration and deceleration event counts as minimum threshold values increased from 0.1 to 3.0 m·s⁻² across 54 elite wheelchair basketball athlete-games using athlete-secured sensors. Each line represents an individual athlete-game. Vertical dashed lines indicate the acceleration (±1.0 m·s⁻²) and high-intensity acceleration (±2.0 m·s⁻²) thresholds used for event classification.


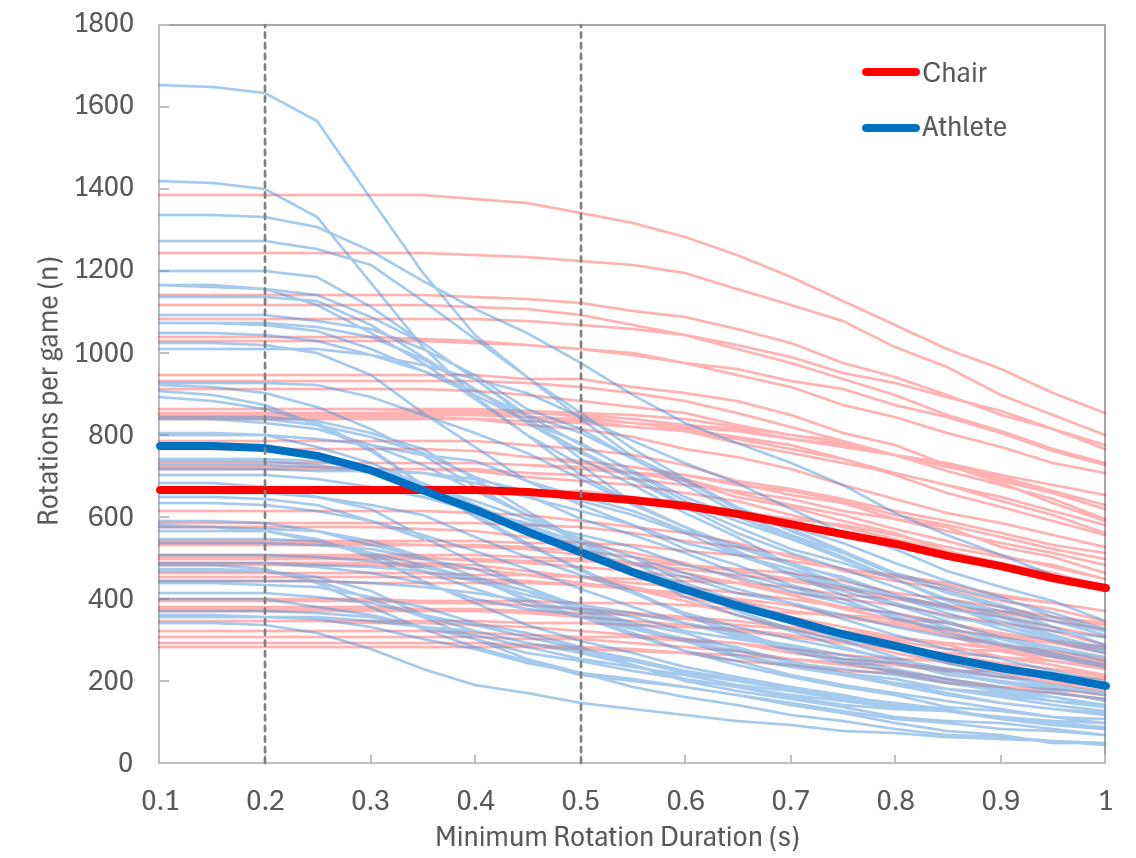


**Supplementary Figure S2.** Sensitivity analysis showing the number of rotations detected when applying minimum rotation duration thresholds ranging from 100 to 1000 ms across 54 elite wheelchair basketball athlete-games. Counts are shown for wheelchair-secured sensors (red) and athlete-secured sensors (blue). Larger solid lines indicate group means, while thinner lines represent individual athlete-game data. Vertical dashed lines indicate the 200 ms threshold used in the present study and the 500 ms threshold previously applied in wheelchair basketball research using wheelchair-secured sensors. All events met the criteria of ≥30° rotational displacement and ≥10°·s⁻¹ angular velocity maintained throughout the rotation.
